# Molecular epidemiology of coagulase-negative *Staphylococcus* species isolated at different lactation stages from dairy cattle in the United States

**DOI:** 10.7717/peerj.6749

**Published:** 2019-05-06

**Authors:** Stephen N. Jenkins, Emmanuel Okello, Paul V. Rossitto, Terry W. Lehenbauer, John Champagne, Maria C.T. Penedo, Andréia G. Arruda, Sandra Godden, Paul Rapnicki, Patrick J. Gorden, Leo L. Timms, Sharif S. Aly

**Affiliations:** 1 Veterinary Medicine Teaching and Research Center, School of Veterinary Medicine, University of California, Davis, Tulare, CA, United States of America; 2 Department of Population Health & Reproduction, School of Veterinary Medicine, University of California, Davis, CA, United States of America; 3 Veterinary Genetics Laboratory, University of California, Davis, CA, United States of America; 4 Department of Veterinary Preventive Medicine, College of Veterinary Medicine, The Ohio State University, Columbus, OH, United States of America; 5 Department of Veterinary Population Medicine, University of Minnesota, Saint Paul, MN, United States of America; 6 Veterinary Diagnostic and Production Animal Medicine, Iowa State University, Ames, IA, United States of America; 7 Department of Animal Science, College of Agriculture and Life Sciences, Iowa State University, Ames, IA, United States of America

**Keywords:** Coagulase negative *Staphylococcus* (CNS), Pulse-field gel electrophoresis (PFGE), Phylogeny, Genetic relatedness, Epidemiology, Mastitis pathogens

## Abstract

**Background:**

Coagulase negative *Staphylococcus* (CNS) species are currently the most prevalent intra-mammary pathogens causing subclinical mastitis and occasional clinical mastitis or persistent infection in lactating dairy cattle. More than 10 CNS species have been identified, but they are generally managed as one group on most dairies in the United States. However, improved management decisions and treatment outcomes may be achieved with better understanding of the prevalent species, pathogenicity and strain diversity within and across dairies.

**Methodology:**

A total of 604 CNS isolates were cultured from milk samples collected during a dry-cow treatment clinical trial conducted on 6 dairy herds in 4 states in the US. All the study cows were randomized to receive 1 of the 3 different intra-mammary antimicrobial infusions (Quatermaster, Spectramast DC or ToMorrow Dry Cow) at dry-off. Milk samples were collected at dry-off, calving (0–6 days in milk, DIM), post-calving (7–13 DIM) and at mastitis events within the first 100 DIM. The CNS isolates were identified to species level by partial sequencing of the *rpoβ* gene, and genetic relatedness within species was investigated by phylogenetic analysis of the pulse-field gel electrophoresis profiles of the isolates.

**Results:**

The major CNS species identified were *S. chromogenes* (48.3%), *S. haemolyticus* (17.9%), *S. simulans* and *S. epidermidis* (each at 6.5%). Other CNS species identified at lower frequencies included *S. hominis*, *S. auricularis*, *S. sciuri*, *S. spp* KS-SP, *S. capitis*, *S. cohnii*, *S. warneri*, *S. pasteuri, S. xylosus, S. hyicus, S. equorum, S. microti, S. rostri, S. gallinarum, S. saprophyticus* and *S. succinus*. Phylogenetic analyses of the major species types demonstrated an association between genetic relatedness and epidemiological distributions of *S. chromogenes, S. simulans, S. haemolyticus* and *S. auricularis.* Additionally, identical strains of *S. chromogenes* and *S. simulans* were isolated from the same udder quarter of several cows at consecutive sample stages. The rest of the minor species had no deducible genetic-epidemiological link.

**Discussion:**

The observed association between genetic and epidemiological distributions indicated animal-adapted nature of four CNS species, suggesting possible host-adapted and environmental transmission of these species. Multi-stage isolation of the same udder quarter strain was evidence for chronic intra-mammary infection.

**Conclusion:**

The different CNS species and strains circulating on US dairy herds were genetically diverse. Four species identified were likely udder-adapted pathogens, 2 of which caused persistent infection. Our findings are important in guiding the design of effective mastitis control strategies.

## Introduction

Mastitis is considered the most prevalent disease in dairy cattle and is endemic in all dairies (USDA-APHIS, 2016). A survey by the National Animal Health Monitoring System (NAHMS) revealed that at least 43% of bulk tank milk samples from dairies tested positive on bacterial culture for *Staphylococcus aureus*, *Streptococcus agalactiae*, or *Mycoplasma* spp (USDA-APHIS, 2008). Similarly, environmental mastitis pathogens were isolated from more than half of the sampled herds that performed individual cow cultures (USDA-APHIS, 2008).

Coagulase negative *Staphylococcus* (CNS) species, variously referred to as non-*aureus* staphylococci (NAS), are currently the most prevalent intra-mammary pathogen of lactating dairy cattle, and as many as 10 different species have been identified in this group (Tenhagen et al., 2006; Thorberg et al., 2009; Condas et al., 2017). The most common isolates are *S. chromogenes*, *S. epidermidis*, *S. simulans*, *S*. *hyicus*, *S. xylosus*, *S. warneri*, and *S. equorum*. Prevalence of CNS in US and European dairy herds ranges from 27% to 55% (Gillespie et al., 2009; De Visscher et al., 2016). Intra-mammary infection (IMI) with CNS is generally associated with subclinical mastitis that may result in increased somatic cell count, and occasional clinical mastitis or persistent infection that leads to reduced milk production (Pyörälä & Taponen, 2009; Tomazi et al., 2015). Increased somatic cell count (SCC) is the main effect observed during CNS intramammary infection (Tomazi et al., 2015). In spite of the increased SCC, some studies have reported slight but significant increase in milk production in CNS infected cows compared to culture negative cows (Schukken et al., 2009). This observation was replicated in an experimental challenge of six primiparous cows with *S. chromogenes* post calving. Following challenge, the cows were infected and showed signs of mild clinical mastitis. Five cows cleared the infection within a few days and the SCC level normalized in 7 days, and one cow developed persistent infection (Simojoki et al., 2009). Generally, CNS organisms are commonly grouped into one class for convenience on most US dairies, and these infections can go untreated or treated without regard to pathogenicity or species isolated (Cameron et al., 2014; Vakkamäki et al., 2017). However, improved management strategies can be achieved with better understanding of the prevalent species and strain diversity within and across dairies (De Visscher et al., 2015). In a recent study, *S. chromogenes* was shown to be the major CNS species causing intra-mammary infection (IMI) in Belgian dairy herds, whereas the remaining CNS species seemed to be environmental in nature since colonization of the teat ends did not increase the odds of IMI (De Visscher et al., 2016). Unlike most mastitis pathogens that can be characterized by phenotypic methods, CNS species may be challenging to identify using colony morphology. Hence accurate identification is dependent on genotypic methods such as pulse-field gel electrophoresis (PFGE), PCR-RFLP (Polymerase Chain Reaction-Restriction Fragment Length Polymorphism) and partial gene sequencing (Zadoks & Watts, 2009). Genetic methods are specifically useful in epidemiological studies to unravel pathogen ecology, infection patterns; for instance, in acute versus chronic infections, and refractoriness of infectious pathogens to treatment such as post-calving IMI (Vanderhaeghen et al., 2015). In PFGE, the total genomic DNA is restricted by endonuclease that cleaves infrequently, and the fragments are analyzed by gel-electrophoresis with alternating power and direction of the electric current. Analysis using PFGE enables simple, accurate and reproducible comparison of isolates for genetic relatedness (Tenover et al., 1995) and several literature reports have documented the use of PFGE in typing staphylococcal species from bovine udder (Mørk et al., 2012; Pate et al., 2012; Bexiga et al., 2014).

Recently, a multi-state clinical trial investigating the efficacy of intra-mammary dry-cow therapy was completed and generated a large repository of CNS isolates (Arruda et al., 2013) suited for a longitudinal study of CNS species-specific IMIs in lactating dairy cattle. Utilizing this collection of isolates, the objective of the study was to describe the molecular epidemiology of CNS species isolated from a cohort of dairy cattle that were randomized to receive one of three commercial intra-mammary dry-off treatments and investigate the strain relatedness of the prevalent CNS species at dry-off, calving, post-calving and mastitis events in the first 100 days post-calving. An additional objective was to determine chronicity of CNS infection for the different species.

## Methods

### Study population

Institutional Animal Care and Use Committee (IACUC) approval was obtained from University of Minnesota, Iowa State University and University of California Davis (protocol number 16313; approval date January 27th, 2011) for a dry cow treatment clinical trial which provided the isolates for the current study (Arruda et al., 2013). The dry cow treatment clinical trial was conducted on six dairy herds in four states, California (dairies CA1 and CA2), Wisconsin (dairies WI1 and WI2), Minnesota (dairy MN) and Iowa (dairy IA), between February 2011 and November 2011. Herds included in the study were enrolled in DHIA testing and willing to comply with the study protocol. The herd sizes ranged between 1,050 and 3,600 lactating cows (average 2,230). The average bulk tank SCC was 242,170 cells/mL (148,000–330,000 cells/mL) and the rolling herd average was 12,360 kg (10,610–13,550 kg). Routine mastitis control practices on all the herds included use of internal teat sealants at dry off, blanket dry cow therapy and commercial coliform mastitis vaccinations. All the study cows were randomized to receive 1 of 3 commercial intra-mammary infusions (Quatermaster, Spectramast DC or ToMorrow Dry Cow) at dry-off, following the manufacturer’s label directions. Individual quarter milk samples were collected according to the guidelines for aseptic milk sample collection procedures (National Mastitis Council, 1999), from 1,091 healthy cows with visibly normal milk at dry off (S1). Study cows were followed up to 100 DIM and individual quarter samples were collected at calving (S2; 0–6 days in milk, DIM), post calving (S3; 7–13 DIM) and at any clinical mastitis (CM) event during the first 100 DIM of the enrolled cows’ subsequent lactation. Gram-positive cocci were identified as CNS if they were catalase positive and coagulase negative (Arruda et al., 2013). A CNS IMI was identified only if two or more morphologically identical colonies were isolated from a 0.01 mL milk sample plated on half plates (Dohoo et al., 2011; Arruda et al., 2013). If a plate had growth of two presumptive *staphylococcus* species, a random pick of one colony from each morphological type was selected. Samples which had growth of more than two morphological distinct colony types were considered contaminated.

Of the 1,091 cows enrolled in the non-inferiority randomized clinical trial, a total of 775 cultured positive in at least one quarter and a total of 3,623 quarter-level intra-mammary pathogens were identified, of which 1,764 were presumptively CNS species by culture and biochemical tests. Majority of these presumptive CNS species isolates were isolated at dry off (702), calving (529) or 5 days post calving (525) and only eight isolates were from clinical mastitis cases (Table 1). From this total, the 211 CNS isolates from the smallest herd (WI2) were excluded from further analysis due to funding limitation. In addition, an approximate 25% proportionate random sample, *n*, across all the sampling stages S1, S2, S3 and clinical mastitis events of all CNS isolates, *N*, from the two largest herds (CA1, *N* = 509, *n* = 125; and IA, *N* = 525, *n* = 127) were selected for further speciation by DNA sequencing and PFGE typing, thereby excluding a total of 993 isolates. Of the remaining 771 CNS isolates, 31 isolates were not submitted for sequencing either due to failure to regrow the organisms from cold storage, the sample was not located at one of the study centers or unknown reason (one isolate). Of the remaining 740 isolates, a total of 115 were not submitted for sequencing due to various reasons; isolates cultured non-CNS species (verified by gram stain, colony morphology and or biochemical test), the culture was not pure, isolates’ freezer location were missing, isolates did not amplify on conventional PCR after two attempts or the PCR product had unexpected band sizes.

**Table 1:** Frequency of coagulase-negative staphylococci (CNS) cultures isolated from quarter milk samples from US dairy cattle. The sample cows were enrolled from 6 dairies in 4 different states during February–November 2011 and followed from dry-off up to 100 days in milk. Quarter samples were taken at dry-off (S1), calving (S2), 7–13 d post-calving (S3), and on first clinical mastitis event post-calving. From the 1,091 cows enrolled in the dry-cow intramammary therapy non-inferiority randomized clinical trial, a total of 775 cultured positive in at least 1 quarter for CNS species and generated a total of 1,764 CNS isolates.

Farm		Frequency of CNS isolates					
	**Dry off (S1)**	**Calving (S2)**	**5 days post calving (S3)**	**Clinical mastitis**^a^	**Presumptive CNS**^b^	**Selected (submitted) for speciation**	**Confirmed CNS species**^d^
CA1	248	131	128	2	509	125 (106)^c^	101
CA2	51	55	50	1	157	137 (137)	125
IA	154	198	173	–	525	127 (114)^c^	112
MN	64	48	57	5	174	141 (141)	136
WI1	98	45	45	–	188	127 (127)	127
WI2^e^	87	52	72	–	211	–	–
Total	702	529	525	8	1,764	657 (625)	601

**Notes.**

a Mastitis events monitored during the first 100 days in milk.

b Confirmed using biochemical tests Catalase positive, coagulase negative.

c Random sample (25%) of presumptive CNS cultures selected from all isolates from CA1 and IA due to budget limitations.

d Confirmed using rpo*β* partial sequencing based on a subset of presumptive CNS isolates.

e Further analysis of presumptive CNS cultures was excluded due to budget limitations.

### *rpoB* partial gene sequencing for CNS species identification

The CNS isolates identified using culture and biochemical tests were selected for *rpoβ* partial gene sequencing (Drancourt & Raoult, 2002). Each isolate was passaged twice on sheep blood agar by inoculation and overnight incubation at 37 °C for 18–22 h. After confirming that the culture was pure, 2–3 colonies were selected and inoculated into 5 mL of BHI broth and incubated overnight at 37 °C. From the overnight culture, 100 µL (∼1 × 10^8^ cells) was pelleted at 16,000 × g for 30 s in a microcentrifuge. The supernatant was discarded, and the cell pellet resuspended in 20 µL of lysis buffer (0.25% SDS, 0.05N NaOH) by vortex. The sample was then heated at 95 °C for 5 min on the thermal block. The cell lysate was pulse-spun at 16,000 × g and diluted in 180 µL of distilled H_2_O. The cell debris was removed from the lysate by pelleting at 16,000 g for 5 min. The supernatant was stored at −20 °C until shipped to the Veterinary Genetics Laboratory, UC Davis (Davis, CA) for PCR amplification and gene sequencing. Partial *rpoβ* sequence (899-bp) was amplified with primer pairs 2643F (5′-CAATTCATG GACCAAGC-3′) and 3554R (5′-CCGTCCCAAGTCATGAAAC-3′) (Drancourt & Raoult, 2002). The PCR products (5 ul aliquots) were visualized by agarose gel electrophoresis, with ethidium bromide staining and under UV light, to identify samples that yielded the expected amplicon. Unidirectional sequencing of PCR amplicons was done with primer 2643F using the BigDye™ Terminator v3.1 Cycle Sequencing Kit (Thermo Fisher Scientific, Waltham, MA, USA) according to manufacturer’s recommendations. Sequenced products were separated by capillary electrophoresis on AB 3730 DNA Analyzer (Thermo Fisher Scientific). Electropherograms were analyzed with SeqMan Pro software (DNASTAR Lasergene) to generate sequences with uniform length of 485 bp. The sequences were compared with *rpoβ* sequences deposited in the NCBI (National Center for Biotechnology Information) database using BLAST (Basic Local Alignment Search Tool) program. Species identification was based on >97% sequence similarity match to reference strains.

### Pulse field gel electrophoresis

The PFGE was performed as previously described (CDC, 2013). Briefly, each bacterial isolate was streaked on sheep blood agar (Trypticase™ Soy Agar, remel R01200) and incubated for 24 h at 37 °C. A single colony was used to inoculate 5.0mL pre-warmed BHI (Brain Heart Infusion (remel R452472) and incubated for 18 h in tightly capped 16X100 glass culture tubes (Fisher14-959-35AA) at 37 °C with rocking at 100 rpm. The bacterial culture was diluted in BHI broth to ∼10^8^ cells/mL by spectrophotometry at 610 nm (Shimadzu UV-1201S). Diluted culture (1 mL) was pelleted at 13,000 rpm for 4 min, supernatant was discarded, the pellet re-suspended in 300 µL TE (Tris-EDTA) buffer and incubated at 37 °C for 10 min. Four microliters of lysostaphin (Sigma; 1mg/mL in 20 mM sodium acetate, pH 4.5) and 300 µL of 1.8% agarose solution at 55 °C (SeaKem®) were added to sample and gently mixed by pipetting 5–7 times and quickly filled into 2 plug mold wells (BioRad 1703706). The mold was allowed to set at 4 °C for 4 min and transferred into 3 mL EC Lysis Buffer (6 mM TrisHCl, 1M NaCl, 100 mM EDTA, 0.5% Brij-58, 0.2% Sodium deoxycholate, 0.5% Sodium lauroylsarcosine) in a 50 mL conical tube. After 4 h incubation at 37 °C, the sample was washed 4 times for 30 min in 15 mL TE buffer while rocking at room temperature. Plugs were stored in TE buffer at 4 °C before cutting out 1.5 mm slices. Slices were equilibrated in 200 µL CutSmart™ buffer (New England Biolabs) at 25 °C for 30 min. The buffer was aspirated off and replaced with fresh 200 µL CutSmart™ buffer containing 1.5 µL Sma1 Enzyme (20,000 U mL^−1^ New England Biolab) and incubated for 3 h at 25 °C before it was aspirated off and replaced with 150 µL of 0.5X TBE buffer and allowed to equilibrate for 15 min. A single slice was used in the electrophoresis (CHEF-DR® III electrophoresis cell; Bio-Rad, Hercules, CA, USA) on 0.5X TBE for 21 h with 120 °C linear ramping of 200 V for 5 to 40 s. The gel was stained with 1% ethidium bromide and image captured with Fluor-S™ MultiImager (BIO-RAD). Images were processed and uploaded on BioNumerics 7.1 (Applied Math) for fingerprint analysis. Spectral analysis was performed, and adjustments were made for Wiener cutoff scale and background as percentages. Normalization was performed using lanes from *Camphylobacter jejuni* as described by Hunter et al. (2005). Similarity coefficient was evaluated using Dice (Zhang et al., 2012), and optimized at 0.5% with a tolerance of 1%. These settings were used for subsequent analysis and dendrogram clustering was achieved by unweighted pair group method with arithmetic means (UPGMA) (Piessens et al., 2011). Phylogenetic trees were generated to assess the similarity of isolates of a specific species from the same cow and udder quarter isolated at dry-off and post calving. Isolates were identified using alphanumeric system starting with herd and state identification number (ID) (California herd 1, CA1; California herd 2, CA2; Iowa herd, IA, Wisconsin herd 1, WI1; Wisconsin herd 2, WI2, Minnesota herd, MN), followed by the sample type (dry-off = S1, calving = S2, 5 days after calving S3, clinical mastitis = CM), species *S. chromogenes* (ch) and *S. haemolyticus* (ha), the unique internal laboratory identification number, the quarter affected (LF, RF, LR, RR) and cow ID.

## Results

### Distribution of CNS species

Based on *rpoβ* sequencing results, 24 (3.8%) presumptive CNS isolates were identified as *Macrococcus* (20) or *S. aureus* (four) and were removed from further analysis, leaving 601 isolates. The frequencies of the different CNS species by dairy and state are shown in Table 2. Overall, *S. chromogenes* was the predominant CNS species across all dairies and states (48.3% of total isolates) followed by *S. haemolyticus* (18.0%), *S. simulans* and *S. epidermidis* (both at 6.5%), *S. hominis* (4.7%), *S. auricularis* (4.2%), *S. sciuri* (2.2%), *S. devriesei* (2.0%), *S. capitis* (1.8%), *S. cohnii* (1.5%) and *S. warneri* (1.0%). The rest of the CNS species identified (*S. pasteuri, S. xylosus, S. hyicus, S. equorum, S. microti, S. rostri, S. gallinarum, S. saprophyticus* and *S.  succinus*) occurred at frequencies less than 1%. Among the predominant species detected, *S. chromogenes* had the highest frequency in all the dairies except WI1 where *S. haemolyticus* had the highest frequency, and *S. simulans* was not detected in CA dairy herds. MN had the highest diversity of CNS species, registering 16 out of the total 20 different species identified in this study, whereas WI had the least diversity of species detected. Species distribution on both CA dairies was the most homogenous, with a majority 77% and 74% *S. chromogenes* detected on CA1 and CA2, respectively, compared to the rest of the dairies that had less than 50% *S. chromogenes*.

### Pulse-field gel electrophoresis

PFGE was performed on 474 CNS isolates out of the total 601 confirmed. The selected isolates were from species that occurred at high frequency and included *S. chromogenes, S. cohnii, S. haemolyticus, S. hominus, S. sciuri, S. epidermidis, S. simulins, S. auricularis,* or *S. capitis*. The other CNS species isolates that were identified at low frequency, specifically *S. equorum, S. gallinarium, S. hyicus, S. microti, S. pasteuri, S. rostri, S. saprophyticus, S. sciuri, S. devriesei, S. succinus, S. warneri* and *S. xylosus* where were not analyzed by PFGE due to funding limitations. In addition, there was unsuccessful PFGE testing attempts on 55 isolates, repeated between one and six times, due to non-characteristic colony morphology on growth, difficulty in re-culturing, difficulty in extracting DNA, non-analyzable bands or no bands. Table 3 outlines the isolates that were strain typed by PFGE.

**Table 2:** Distribution of coagulase-negative *Staphylococcus* (CNS) species isolated from milk samples from cattle on 5 US dairies. The milk samples were taken at dry-off, calving, 7 days post-calving and during mastitis events occurring within 100 days in milk (DIM). All isolates were identified by bacterial culture, coagulase and catalase testing, and confirmed by *rpoB* partial gene sequencing.

Organism	Dairy ID (State abbreviation and dairy number)						
	CA1	CA2	IA	MN	WI1	Total	Percent total
*S. chromogenes*	**78**	**92**	**44**	**34**	**42**	290	48.3
*S. haemolyticus*	8	5	20	24	51	108	18.0
*S. simulans*	–	–	12	21	6	39	6.5
*S. epidermidis*	5	12	5	12	5	39	6.5
*S. hominis*	–	1	11	2	14	28	4.7
*S. auricularis*	–	1	6	15	3	25	4.2
*S. sciuri*	2	4	4	1	2	13	2.2
*S. capitis*	–	5	1	5	–	11	1.8
*S. cohnii*	–	1	3	3	2	9	1.5
*S. warneri*	2	–	–	4	–	6	1.0
*S. pasteuri*	–	2	1	2	–	5	0.8
*S. xylosus*	–	–	1	3	–	4	0.7
*S. hyicus*	1	2	–	–	–	3	0.5
*S. equorum*	1	–	1	–	–	2	0.3
*S. microti*	2	–	–	–	–	2	0.3
*S. rostri*	1	–	–	1	–	2	0.3
*S. gallinarum*	1	–	–	–	–	1	0.2
*S. saprophyticus*	–	–	–	1	–	1	0.2
*S. succinus*	–	–	–	1	–	1	0.2
*S. spp KS-SP*	**–**	**–**	3	7	2	12	2.0
Total	101	125	112	136	127	601	100.0

**Table 3:** Distribution of CNS species analyzed using PFGE by State and Farm.

Organism	Dairy ID (State abbreviation and dairy number)					
	CA1	CA2	IA	MN	WI1	Total
*S. chromogenes*	76	83	37	30	39	**265**
*S. haemolyticus*	8	5	19	20	49	**101**
*S. simulans*	–	–	10	21	6	**37**
*S. hominis*	–	1	9	2	10	**22**
*S. epidermidis*	2	5	3	4	3	**17**
*S. auricularis*	–	1	5	5	–	**11**
*S. sciuri*	2	2	3	1	2	**10**
*S. capitis*	–	2	1	3	–	**6**
*S. cohnii*	–	–	2	1	2	**5**
Total	88	99	89	87	111	**474**

#### Phylogenetic analysis for genetic relatedness of isolates

Phylogenetic trees were constructed for each CNS species to compare genetic relatedness of the isolates based on PFGE banding patterns. The trees were constructed using the UPGMA algorithm as a simple agglomerative hierarchical clustering method to show similarity between the isolates. The tree analysis showed that 100% similarity score occurred between isolates with the same number and pattern of the bands, and were designated as genetically indistinguishable (Fig. S1). Isolates with >80% similarity score had a difference of 2–3 bands in the banding pattern or band size, attributed to one genetic event, and were interpreted as closely related strains. A similarity score of >50% between isolates was interpreted as possibly genetically related and constituted a clade. Within a clade, there were 4–6 band differences consistent with 2 separate genetic events. Organisms with <50% similarity were considered genetically unrelated.

**Figure 1:**
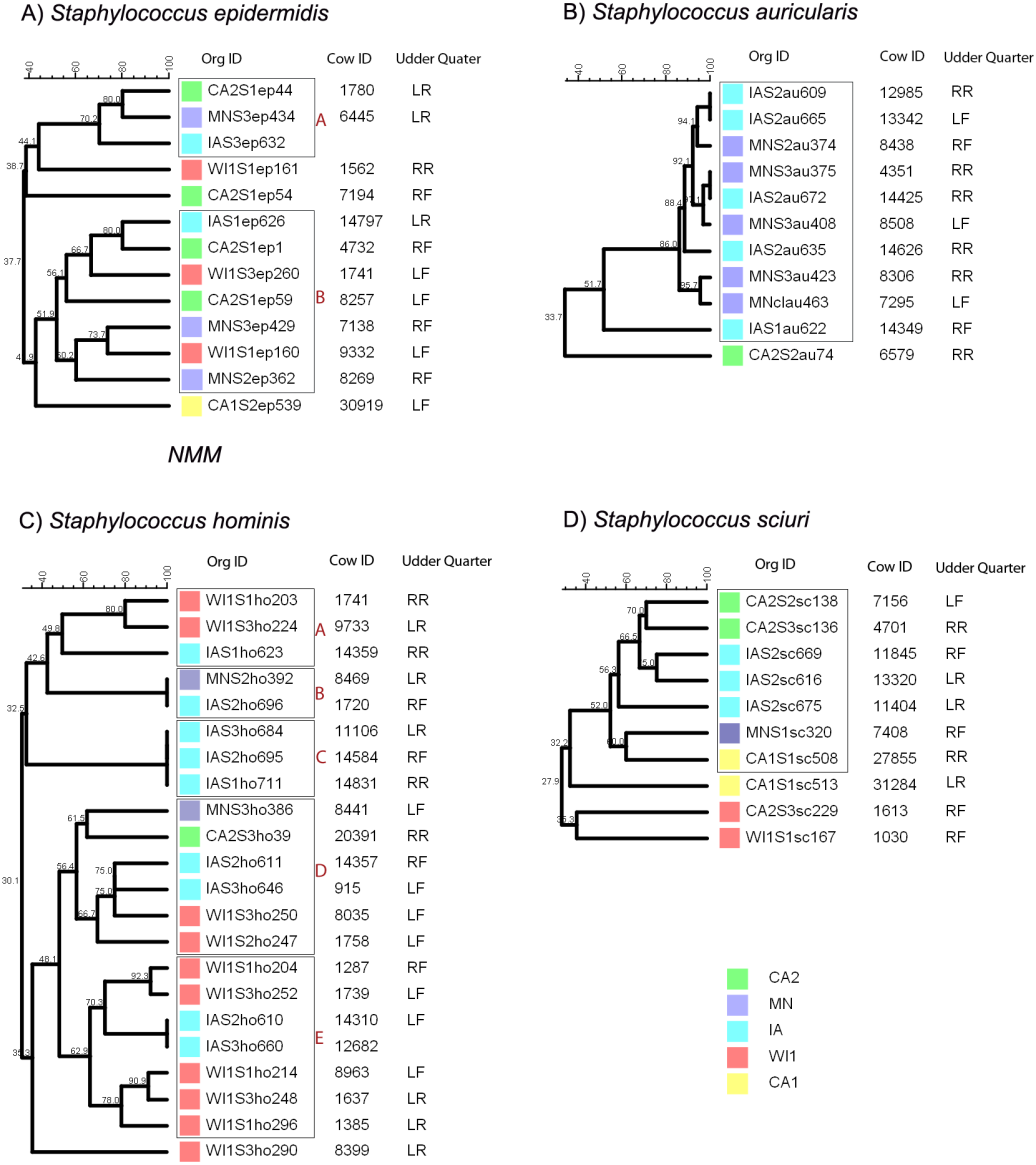
Genetic relatedness of coagulase negative *Staphylococcus* spp. (CNS) isolated from US dairies. The unweighted pair group method with arithmetic mean (UPGMA) phylogenetic genetic trees were constructed based on PFGE banding patterns. Similarity scores are shown at the tree branches. (A) *Staphylococcus epidermidis* strains clustered into two clades (A and B) and three distinct isolates. The isolates showed wide genetically diversity and heterogenous distribution between the five dairies. (B) *Staphylococcus auricularis* strains had least genetic diversity among the isolates. The isolates clustered into 1 clade, except for a single isolate from a California dairy. The rest of the isolates were from Midwestern dairies. Within the clade, most of the isolates had close genetic relationship (>80% similarity) and two isolate pairs were identical (100% similarity). (C) *Staphylococcus hominis* strains had wide genetic diversity and clustered into 5 clades (A, B, C, D and E) and a single distinct isolate. Clade A had two closely related isolates from WI and a possibly related isolate from IA. Clade B contained identical isolates from two MN and IA. Clade C had three identical isolates from IA. Clade D was the most diverse with isolates from all the states (CA, IA, MN and WI). Clade E contained identical isolates from IA and the rest of the isolates were from WI. (D) *Staphylococcus sciuri*. *S. sciuri* clustered into 1 clade and 3 distinct isolates. The group had wide genetic diversity and none of the isolates were closely related. Dairies: CA1, California 1; CA2, California2; IA, Iowa; MN, Minnesota; WI or WI1, Wisconsin1. Udder quarters: RF, Right front; LF, Left front; RR, Right rear; LR, Left rear.

*Staphylococcus epidermidis* group was genetically diverse with a maximum of 80% similarity between all the different isolates (Fig. 1A). The strains clustered into two clades and three distinct isolates. Within each clade, all the strains were heterogenous with no observable correlation between the genetic relatedness and the dairy of origin.

*Staphylococcus auricularis* group had the least genetic diversity among all the different CNS species (Fig. 1B) and clustered into a single clade comprised of isolates from the IA and MN. One strain that was distinctly unrelated to the rest of the isolates (33.7% similarity) was isolated from CA2. Within the single clade, 90% of the isolates had close genetic relationship with >80% similarity, and 1 isolate pair from IA dairy was genetically indistinguishable (100% similarity).

*Staphylococcus hominis* clustered into five distinct clades and a single distinct isolate as shown in Fig. 1C. Within each clade, the *S. hominis* had close genetic relationship, especially within a dairy, and majority of isolates from dairy MN were genetically indistinguishable. Clade A was comprised of two closely related isolates from WI and a possibly related isolate from IA. In contrast, clade B contained genetically indistinguishable isolates from two separate dairies (MN and IA). Similarly, all the isolates in clade C were genetically indistinguishable and were all from IA. Clade D was the most diverse with isolates from all the states (CA, IA, MN and WI). Within clade E, two isolates from IA were genetically indistinguishable, and the rest of the isolates from WI formed two closely related subgroups. One isolate from WI1 was distinctly unrelated to all the other groups.

*Staphylococcus sciuri* strains had wide genetic diversity and distribution across all the five premises (Fig. 1D). There was no observed close genetic relationship between any isolates. The isolates clustered into one clade and three distinct isolates. Within the clade, two isolate pairs from CA2 and IA had high similarity scores of 70% and 85%, indicating a probable genetic relationship.

**Figure 2:**
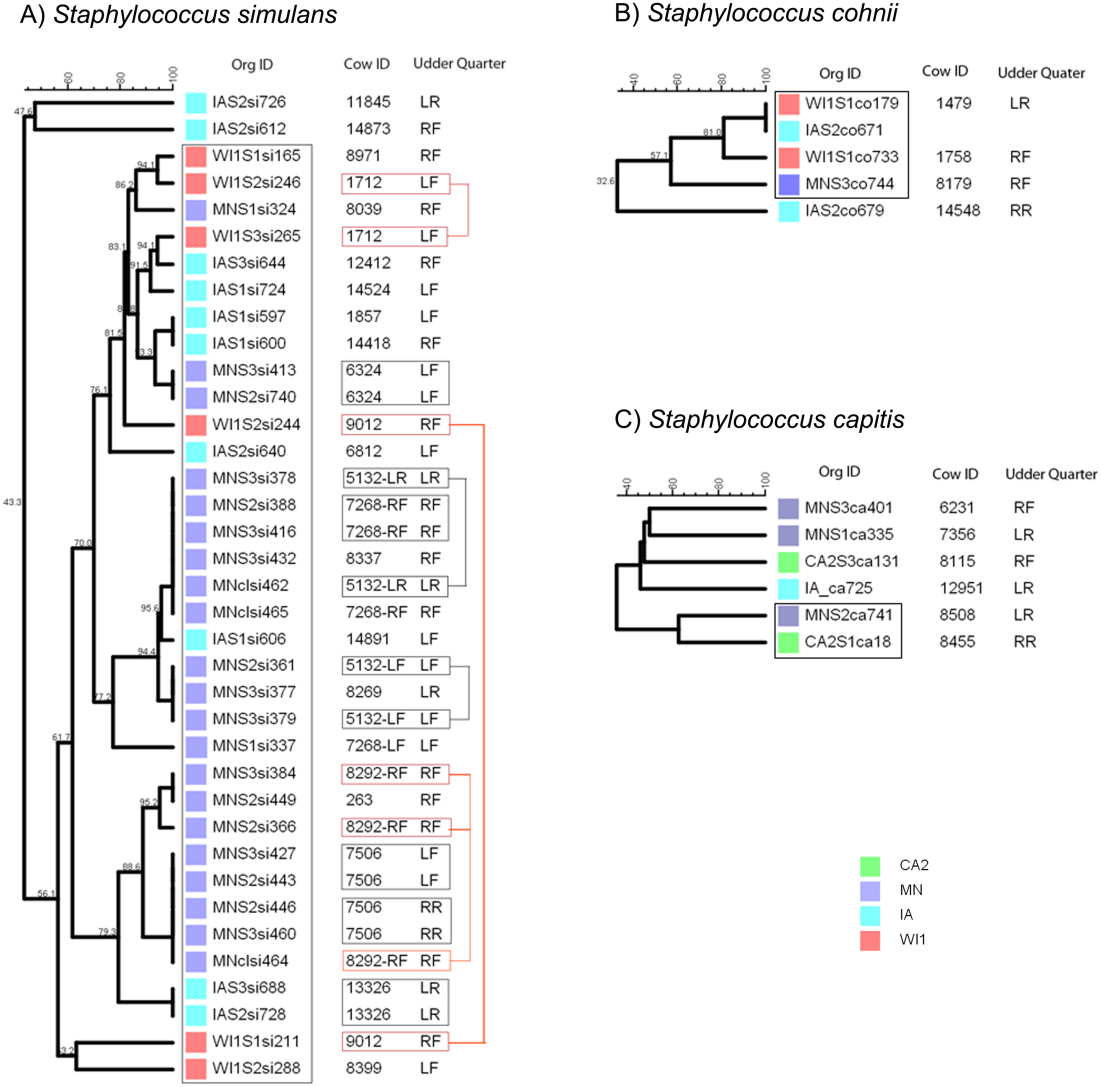
Genetic relatedness of coagulase-negative *Staphylococcus* spp. (CNS) isolated from US dairies. The unweighted pair group method with arithmetic mean (UPGMA) phylogenetic trees were constructed based on PFGE banding patterns. (A) *Staphylococcus simulans*. All the *S. simulans* strains were isolated from Midwestern dairies and had close genetic relationships, constituting one clade and a two distinct strains from IA. Six cows had indistinguishable isolated from the same udder quarter at calving (S2) and 5 days post calving (S3) (black boxes). The S2 and S3 isolates from Cow 1712 and 5132 had close genetic relationship, but the S1 (dry-off) and S2 isolates from Cow ID 9012 were genetically distant (red boxes). (B) *Staphylococcus cohnii* strains clustered into a single clade and one distinct isolate. Two isolates from IA and WI1 were genetically indistinguishable. (C) *Staphylococcus capitis* strains had wide genetic diversity with genetically distinct strains, except two isolates from MN and CA2 that showed possible genetic relationship. Dairies: CA2, California 2; IA, Iowa; MN, Minnesota; WI or WI1, Wisconsin 1. Udder quarter: RF, Right front; LF, Left front; RR, Right rear; LR, Left rear. Similarity scores are shown at the tree branches.

*Staphylococcus simulans* strains comprised a large group of 37 strains that were isolated only from the Midwest dairies (IA, MN and WI1) (Fig. 2A). Most of the strains (35) had close genetic relationships and formed one big clade with 56.1% similarity score, and the remaining two strains were genetically distinct (47.6% similarity). Strains within the clade clustered into smaller sub-clades that roughly corresponded to the dairy of origin. Six study cows in MN and one cow from IA had one or more pairs of genetically indistinguishable *S. simulans* strains isolated from the same quarter at different stages over the study period. Six cows from this group had indistinguishable strains isolated at calving (S2) and 5 days post calving (S3). Strains isolated during the S2 and S3 stages from two other cows in the group had close genetic relationship, but the S1 (dry-off) and S2 isolates from cow 9012 were genetically distant.

*Staphylococcus cohnii* strains comprised the smallest group with 5 isolates from mid-western dairies in IA, MN and WI1 (Fig. 2B). All the isolates grouped into 1 clade except a single distinct isolate from IA (32.6% similar). Within the clade, two isolates from WI1 and IA were genetically indistinguishable, and the pair had close genetic relationship with another isolate from WI1 (81.1% similarity).

*Staphylococcus capitis* strains were a small group of six isolates from MN, CA2 and IA dairies, and had the most genetic diversity (Fig. 2C). Four strains were genetically distinct, and only 2 isolates from MN and CA2 showed possible genetic relationship (72% similarity).

**Figure 3:**
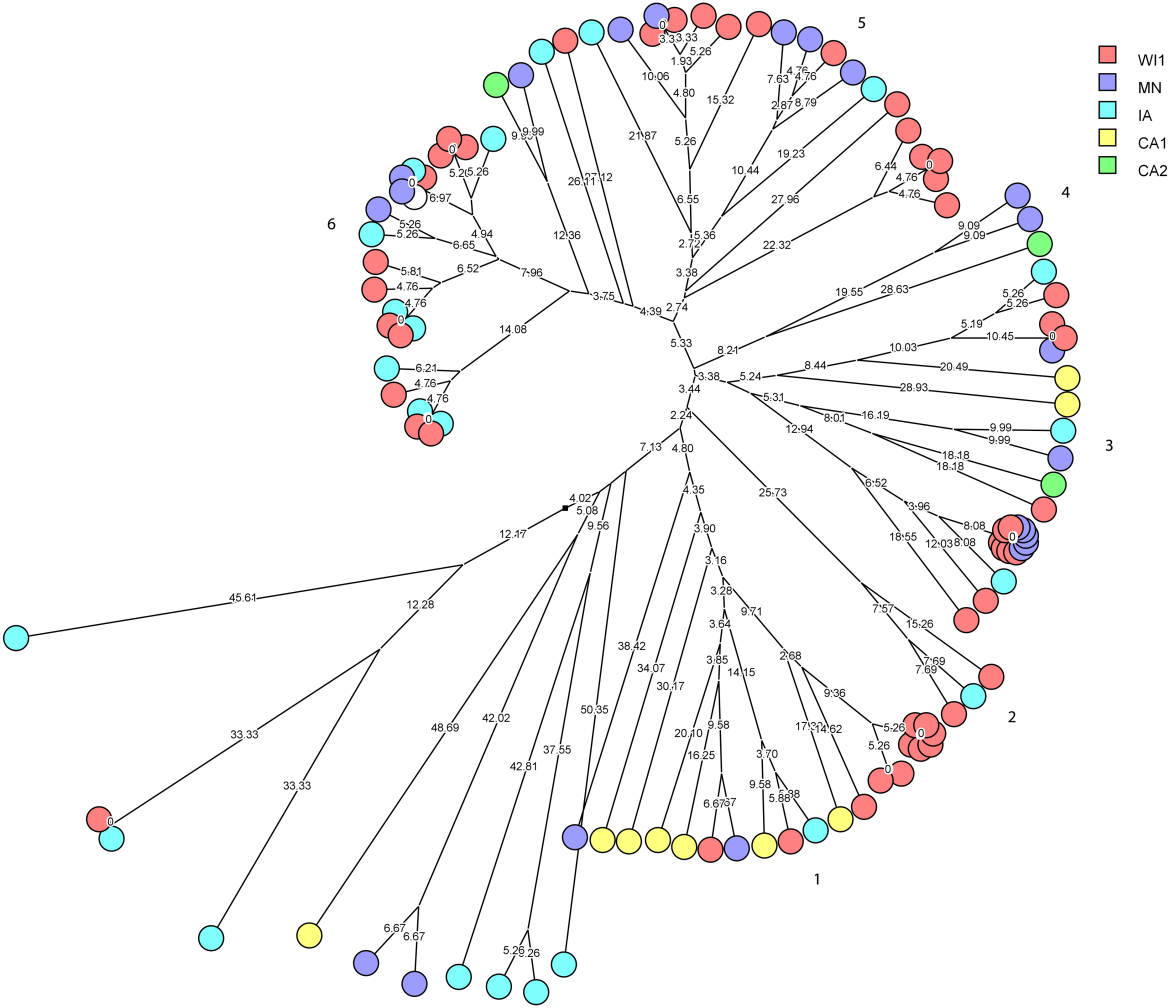
Phylogenetic cluster analysis of *Staphylococcus haemolyticus* strains isolated from US dairies. The multidimensional network was created by agglomeration scheme from unweighted pair group method with arithmetic means (UPGMA) dendrogram based on PFGE banding pattern. The *S. haemolyticus* isolates formed six major heterogenous clusters (1–6) comprised of strains from the different dairies. The branch lengths labels indicate the genetic distance and isolates with zero distance are clustered together at the end node. Dairies: CA1, California 1; CA2, California 2; IA, Iowa; MN, Minnesota; WI1, Wisconsin 1.

*Staphylococcus haemolyticus* was the second largest group of CNS isolates in the study (101 isolates) with wide genetic diversity. We used cluster analysis to generate multidimensional phylogenetic network to facilitate interpretation of relatedness using genetic distance (Fig. 3). The strains clustered into major six groups, and multiple subgroups of closely related isolates within the major groups. The strains within a subgroup were predominantly comprised of isolates from one to three dairies, and several isolates from WI1, MN and IA were genetically indistinguishable. Only one cow had the same strain of *S. haemolyticus* isolated at S2 and S3 stages.

**Figure 4:**
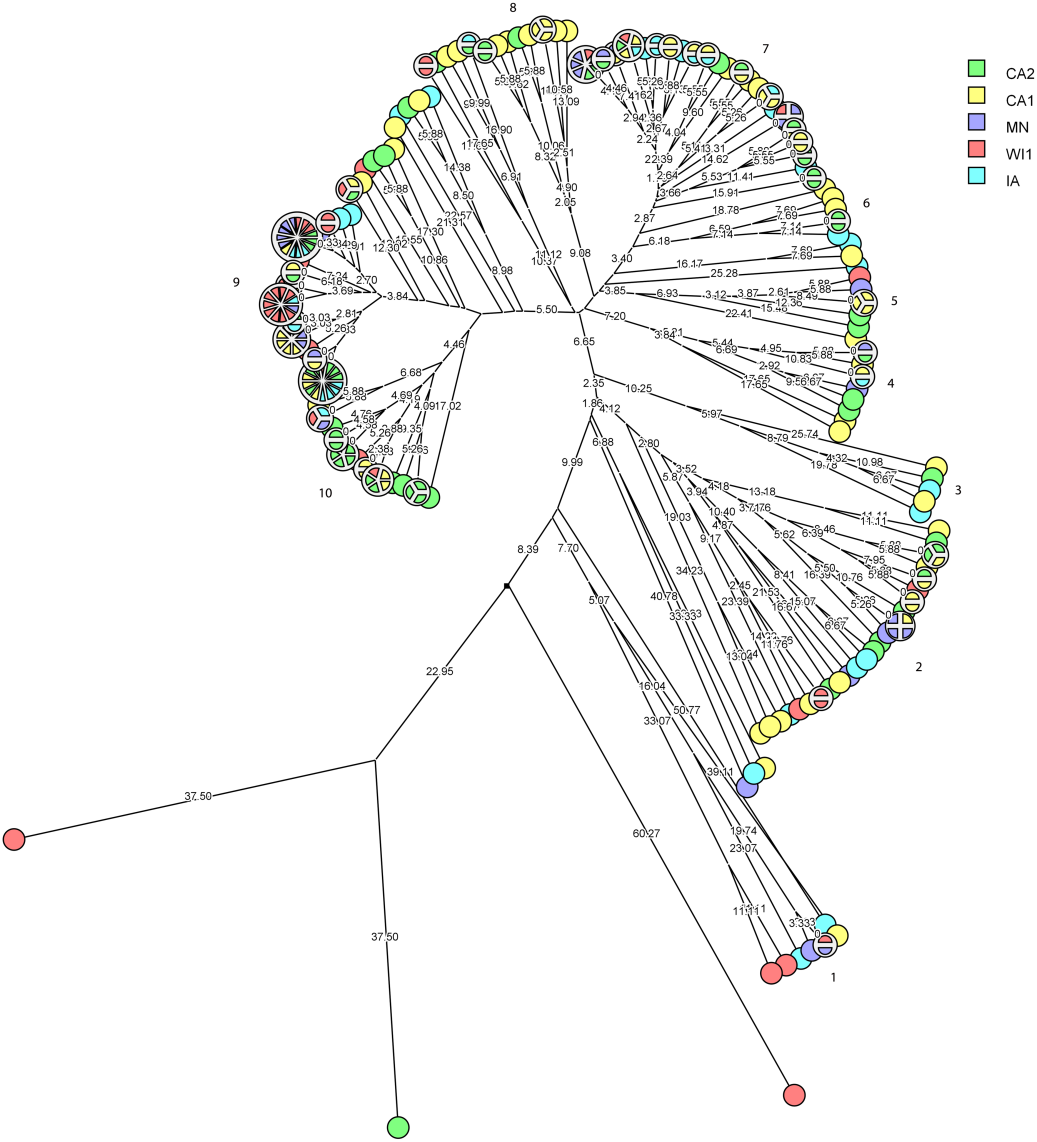
Phylogenetic cluster analysis of *Staphylococcus chromogenes* strains isolated from US dairies. The multidimensional network was created by agglomeration scheme from unweighted pair group method with arithmetic means (UPGMA) dendrogram based on PFGE banding pattern. The isolates formed 10 major heterogenous clusters (numbered 1–10) that encompassed strains from the different dairies. Clusters 9 and 10 had the most number of isolates with close genetic distances or genetically identical isolates. The branch labels indicate genetic distance and isolates with zero distance are clustered together at the node. Dairies: CA1, California 1; CA2, California 2; IA, Iowa; MN, Minnesota; WI1, Wisconsin 1.

*Staphylococcus chromogenes* comprised the largest group of CNS isolates and had wide genetic diversity. Following cluster analysis, the strains formed 10 major groups and several subgroups of closely related strains (Fig. 4). The distribution of the strain types across the dairies was quite heterogeneous with strains from all dairies spread across all the different groups. There were however several groups of isolates with zero genetic distances. The majority of these isolates, however, were repeat isolates from the same cows at different sampling stages. The repeat isolates were selected for analysis of persistence, defined as close relatedness of strains isolated from the same cow at different stages over the study period.

**Figure 5:**
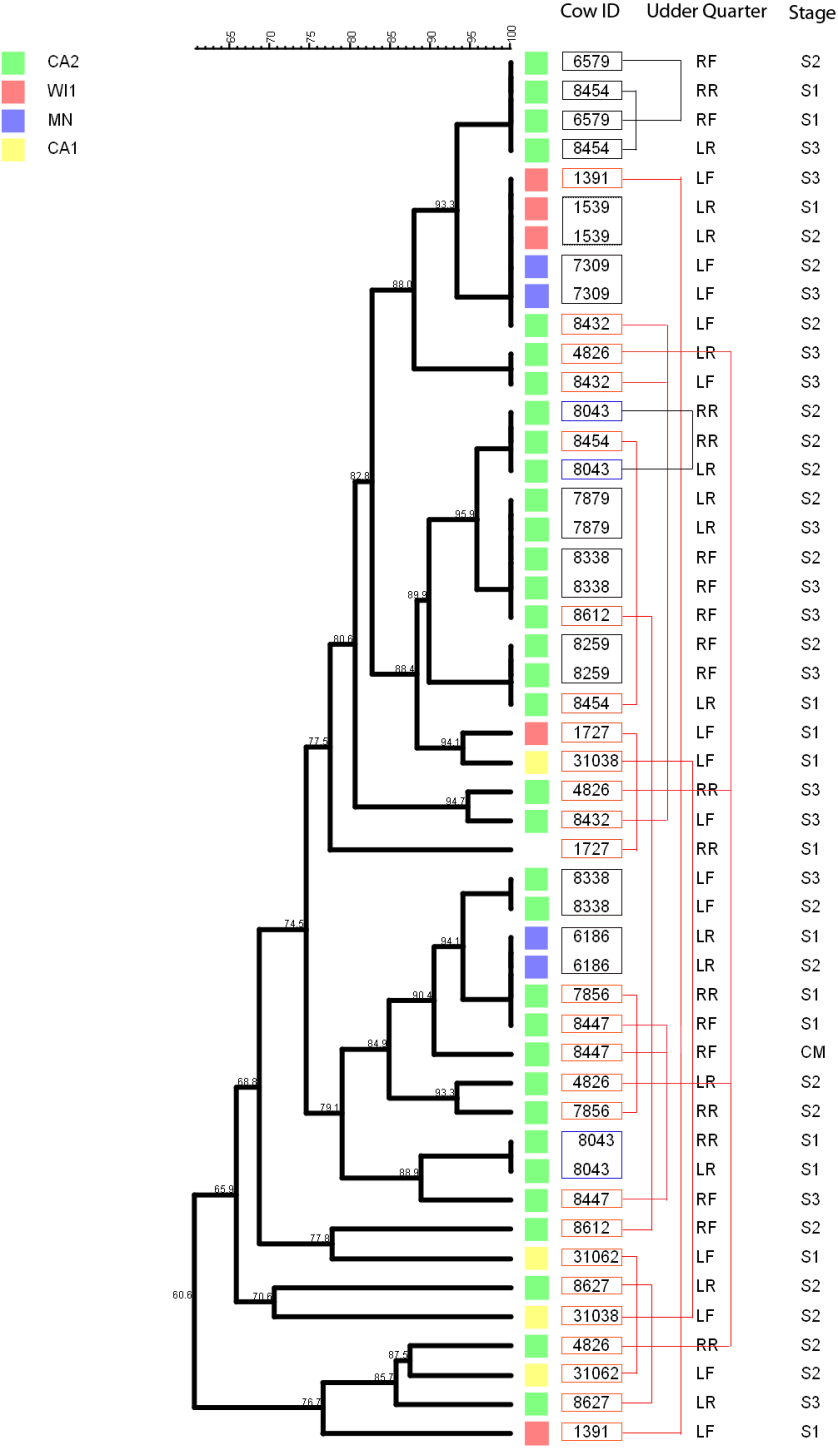
Persistence of *Staphylococcus chromogenes* intramammary infection in lactating cows on US dairies. The phylogenetic tree for the repeat isolate strains (*n* = 48) was created using unweighted pair group method with arithmetic means (UPGMA) method, based on PFGE band patterns. The strains were isolated from quarter milk samples at dry off (S1), calving (S2), 5 days post calving (S3) and at any clinical mastitis event (CM) within 100 days in milk (DIM). Eight cows (black boxes) had genetically indistinguishable strains isolated from the same udder quarter at two different stages (three cows had S1/S2 each, one cow had a S1/S3 isolate pair, and three cows S2/S3 isolates each, and one cow had 2 S2/3 isolates from different quarters). Two cows each had a pair of genetically indistinguishable strains isolated from different quarters at the same sample stage (blue boxes). Twelve cows had related strains isolated from 1 or 2 udder quarters at the same stage or different stages (red boxes and connecting red lines). Only one cow had CM isolate that was closely related to S1 isolate and possibly related to S3 isolate of the same quarter. Dairies: CA1, California 1; CA2, California 2; MN, Minnesota; WI1, Wisconsin 1. Udder quarters: LF, Left Front; RF, Right Front; RR, Right Rear; LR, Left Rear.

We analyzed a subset of *S. chromogenes* (Fig. 5) for persistence by determining the genetic relatedness of 48 isolates from cows with multiple isolates over the different sampling stages. This group comprised of strains isolated from either the same or different quarter milk samples at dry off (S1), calving (S2), 5 days post calving (S3) and during a clinical mastitis (CM) event occurring within 100 DIM. Ten cows had genetically indistinguishable isolate pairs from the same quarter at different stages. Most of these cows were from CA2 dairy. The isolate pairs were distributed across the study with three pairs isolated at S1–S2 stage, five pairs from S2 to S3, and one pair each from different quarters at S1 and S2 stages. Additionally, 26 possibly related strains were isolated from each of the 12 different cows over two different study stages or same stage but different quarters. A total of three, four and two strains were from the same quarters at S1–S2, S2–S3 and S1–S3 stages, respectively. Three isolate pairs were from the same stage (S1, S2 or S3) but from different quarters. In one cow, there were three different isolates from the same quarter at S1, S2 and S3 stages.

## Discussion

In this longitudinal study, *rpoβ* partial gene sequence and PFGE analyses were used to characterize the molecular epidemiology of CNS species causing IMI on US dairies located in the Midwest and California. To the best of our knowledge, this is the single most comprehensive study that characterized the species and genetic diversity of CNS strains from several states and dairies in the US. A total of 604 CNS isolates from five dairies across four states were included in the study, and our results showed varied distribution of 20 different CNS species with diverse genetic strains across dairies. Most of the isolates originated from dry off, calving or post calving stages and on a few were form clinical mastitis cases. The large sample size allowed wide range comparison of species and strain distributions, and to draw conclusions on host-associated transmission pattern for three CNS species including *S. auricularis,* which had not been previously reported. In addition, we showed evidence of persistent subclinical IMI caused by two other species, *S. simulans* and *S. chromogenes.*

The CNS species were identified by partial sequencing of *rpoβ* gene, a tool developed for identification of *Staphylococcus* species (Drancourt & Raoult, 2002) that has been used as a reference to validate other identification methods (Spanu et al., 2011). By *rpoβ* sequencing, 24 initial presumptive CNS isolates were correctly identified as *Macrococcus* species or *S. aureus* and were dropped from further analyses, indicating the high discriminative power of this method compared to phenotypic or biochemical test methods (Da Silva et al., 2000; Zadoks & Watts, 2009). The overall predominant CNS species on all dairies was *S. chromogenes* followed by *S. haemolyticus,* except in the WI1 herd where this order was reversed. Other CNS species; *S. simulans*, *S. epidermidis*, *S. homini* s and *S. auricularis*, *S. sciuri*, *S. devriesei*, *S. capitis*, *S. cohnii* and *S. warneri* occurred at lower frequencies (1–10%) and the remaining species were of minor frequency at <1%. Our results were partly consistent with previous reports that showed *S. chromogenes* as the predominant CNS isolate from most cases of subclinical mastitis in dairy herds (Sawant, Gillespie & Oliver, 2009; Piessens et al., 2011; Raspanti et al., 2016). There is however significant variation in the prevalence of the major CNS species between herds. *S. haemolyticus* has been reported at a lower frequency in most herds, and *S. epidermidis* has been reported as the second most frequent isolate in herds from the US (Sawant, Gillespie & Oliver, 2009) and Europe (Sampimon et al., 2009). In addition, other minor species such as *S. xylosus* and *S. simulans* have been reported to occur at a higher frequency in other herds compared to our results (Supré et al., 2011; Björk et al., 2014). The variation in CNS species between herds may be attributed to herd-level factors involved in the establishment of a particular species on a dairy (Piessens et al., 2011). The same distribution variations were reflected in our results where the proportions of *S. chromogenes* in the larger dairies from California (CA1 and CA2) were very high (>73%) compared to the low proportions in the relatively smaller herds from the Midwestern US dairies (25–41%). Since different CNS species have been associated with various body sites or environment reservoirs, future studies on management and control strategies should aim at understanding species specific risk for occurrences and transmission at the species and herd level (Piessens et al., 2011).

To deduce the genetic relationship between strains, we used dendrograms based on PFGE banding patterns to demonstrate relatedness. PFGE is a valuable tool that has been previously used to study the diversity and persistence of staphylococcal species in the bovine udder (Mørk et al., 2012; Bexiga et al., 2014). The interpretation of the PFGE bands and strain similarity relied on previously proposed guidelines (Tenover et al., 1995). The dendrograms generated grouped strains within each species into characteristically distinct clusters, which we speculate to indicate probable differences in ecology and transmission patterns at the species level. However, our sample size may not be sufficient to confirm transmission modes. The less frequent species, *S. hominis*, *S. sciuri, S. epidermidis, S. capitis* and *S. cohnii,* had high genetic diversity which was demonstrated by the low similarity scores between the strains. The latter finding partially supports the previous report by De Visscher et al. (2016), which associated *S. sciuri* and *S. cohnii* intra-mammary infection (IMI) at parturition with with dirty teat apices before calving. At the herd level, the same authors demonstrated the effect of seasonality on the occurrence of *S. cohnii* and *S. sciuri* in bulk tank milk samples from different dairies (De Visscher et al., 2017). However, the presence of *S. epidermidis* in bulk tank milk had no significant association with evaluated herd-level factors such as housing and hygiene. These observations agree with our findings and support the environmental nature of these species that are thought to be opportunistic, and the strains have a higher degree of genetic diversity. *S. auricularis* also occurred at a lower frequency but the strains were homogenous with close genetic relationship in 90% of the isolates, and a pair of isolates from the IA were genetically indistinguishable with 100% similarity score. Such similarity may suggest possible host-adapted nature of *S. auricularis*. However, *S. auricularis* was isolated at a low frequency and there was limited literature on its occurrences to support interpretation of our finding.

Although *S. haemolyticus* occurred at the second highest frequency of 62 isolates forming 10 clusters of strains with 100% similarity scores, only two identical isolate pairs were isolated from the same udder quarter at two consecutive stages (S1–S2 and S1–S3). These findings reinforce previous observations that *S. haemolyticus* may be a primarily environmental pathogen and subclinical IMIs are possibly associated with factors such as dirty teat apices (Piessens et al., 2011; De Visscher et al., 2015; Vanderhaeghen et al., 2015; De Visscher et al., 2016). The *S. simulans* strains were the third most frequent species and clustered into groups with close genetic relationships. Several cows had closely related, or genetically indistinguishable strains isolated from the same udder quarters at consecutive sample stages. The same phenomenon was also observed in *S. chromogenes,* the predominant CNS species isolated in this study. Our results support previous observations that *S. chromogenes* and *S. simulans* are the more relevant CNS species that are commonly isolated from milk and body sites such as the udder skin, teat apices and teat canal, mainly from heifers and first lactation cows (Taponen, Björkroth & Pyörälä, 2008; De Visscher et al., 2015; Vanderhaeghen et al., 2015; De Visscher et al., 2016; Adkins et al., 2018). *S. chromogenes* have additionally been isolated from other body sites such as the haircoat and the vagina, indicating the species is adapted to the bovine host (White et al., 1989). Similarly, (Taponen, Björkroth & Pyörälä, 2008), reported isolation of *S. simulans* from mastitis samples and three other isolates from the udder skin, teat apex and the teat canal, with the interpretation that *S. simulans* could have been more specifically associated with mastitis.

In a Belgian study, De Visscher et al. (2017), reported that herds that did not clean milking equipment after subclinical cows have been milked tended to have positive bulk tank results for *S. simulans*, *S. haemolyticus*, or *S. cohnii* which may point at the potential for contagious transmission given substandard milking practices. In this study, more than half of the *S. simulans* strains (53.8%) isolated from the udder quarter or different udder quarters of the same cows at different stages were either genetically indistinguishable or closely related. *S. chromogenes* strains were however more genetically diverse, and only a small proportion of genetically indistinguishable strains or closely related strains were isolated from more the same udder quarter or different quarters of the same cow at consecutive sample stages (S1–S2 or S2–S3). The occurrence of indistinguishable isolates at consecutive may indicate persistent infections. Similarly, the occurrence of closely related strains at different stages may have resulted from persistent infection with genetic mutation of the strains over time. On the other hand, the closely related strains isolated at consecutive sample stages was possibly due to chronic IMI with two different strains selected at the different stages. Generally, these observation suggest that *S. chromogenes* and *S. simulans* are more likely associated with persistent subclinical IMIs compared to other CNS species. The observed genetic similarity of strains across different cows within the same herd also point at the potential for contagious, or both contagious and environmental transmission patterns depending on circumstances that include but may not be limited to milking practices. These observations are supported by a previous report on the possibility of persistent CNS infections by Pyörälä & Taponen (2009). Certain strains from different states were found to be 100% similar using the current PFGE analysis, such as, one pair of *S. hominis* isolates from herds in MN and IA, one pair of *S. auricularis* from IA and MN herds, one pair of *S. cohnii* from WI1 and IA herds, five *S. chromogenes* isolates from CA2, MN and WI1 herds, and four *S. chromogenes* isolates from CA2 and MN herds. Such similarity may be due to limitations in the discriminatory power of PFGE as a DNA finger printing technique, less likely due to laboratory error at sample, culture, PCR, or PFGE testing level, or finally due to coincidence since these may be truly identical strains albeit highly unlikely.

Currently, CNS infections in dairy herds are managed as a single group with no consideration for the different species (Pyörälä & Taponen, 2009; Ruegg, 2012) and most subclinical CNS infections go untreated (Pyörälä & Taponen, 2009). These infections typically cause mild inflammation in the infected quarters resulting in a 3–4 fold increase in the composite somatic cell count (SCC) (Thorberg et al., 2009). With the increasing emphasis currently on lower bulk tank SCC and associated quality premiums, however, CNS IMIs can reduce both milk quality and price, which is an incentive for improved control (Sears & McCarthy, 2003). Compared to *S. aureus*, CNS infections respond to antimicrobial treatment even though resistance to antimicrobials is more common for CNS (Taponen et al., 2003; Pyörälä & Taponen, 2009). Previous work has shown that for both clinical and subclinical mastitis caused by CNS, the bacterial cure rate for quarters treated with antimicrobials was significantly higher at 86% compared to only 46% for untreated quarters (Taponen et al., 2006).

Generally, the majority of the CNS isolates originated from S1, S3 and S3 samples, and only six confirmed CNS species were from clinical mastitis cases (Tables 1 and 4). The very low number of isolates from the clinical mastitis cases shows that CNS has low virulence potential and mainly cause subclinical chronic infections. Our findings point at the relative importance of the different CNS species causing mastitis and possible persistent infections, and therefore, provide a good argument for the need for species identification. Hence, a need exists for the development of rapid and low-cost diagnostic tests for the most frequent and relevant CNS species, and account for the various regional species distributions. Such tests could be biochemical or antibody-based that specifically identify aerobic culture colonies to species level to help increase our knowledge about the important CNS species isolated from clinical samples. In addition, such tests may aid in decision making on specific treatment to eliminate persistent infections, such as *S. chromogenes* and *S. simulans,* or implement control measures such as teat dipping and best milking practices.

**Table 4:** Distribution of coagulase-negative Staphylococcus (CNS) species isolated from milk samples from cattle on five US dairies by sampling stage. Quarter samples were taken at dry-off (S1), calving (S2), 7–13 d post-calving (S3), and on first mastitis event post-calving (CM).

**Organism**	**Sampling stage**				**Total**	**Percent total**
	**S1**	**S2**	**S3**	**CM**		
*S. chromogenes*	144	76	69	1	290	48.3%
*S. haemolyticus*	39	39	30	–	108	18.0%
*S. epidermidis*	17	9	13	–	39	6.5%
*S. simulans*	9	14	13	3	39	6.5%
*S. hominis*	10	8	10	–	28	4.7%
*S. auricularis*	3	10	10	2	25	4.2%
*S. sciuri*	6	5	2	–	13	2.2%
*S. devriesei*	7	2	3	–	12	2.0%
*S. capitis*	5	2	4	–	11	1.8%
*S. cohnii*	5	3	1	–	9	1.5%
*S. warneri*	1	1	4	–	6	1.0%
*S. pasteuri*	2	1	2	–	5	0.8%
*S. xylosus*	–	2	2	–	4	0.7%
*S. hyicus*	–	–	3	–	3	0.5%
*S. equorum*	1	–	1	–	2	0.3%
*S. microti*	–	2	–	–	2	0.3%
*S. rostri*	–	2	–	–	2	0.3%
*S. gallinarium*	1	–	–	–	1	0.2%
*S. saphrophyticus*	–	–	1	–	1	0.2%
*S. succinus*	–	1	–	–	1	0.2%
**Total**	**250**	**177**	**168**	**6**	**601**	100.0%

Although PFGE is a powerful tool in surveillance, limitations of the method have been shown in distinguishing closely related bacterial strains such as *Escherichia coli* O157:H7 (Böhm & Karch, 1992) or methicillin-resistant *Staphylococcus aureus*. The inherent limitation of PFGE could have influenced the interpretation of our results. In addition, we took a random sample of the CNS isolates from two dairies (CA1 and MN) for PFGE analysis, due to budget limitation. The random sampling process may have interrupted the sequence of isolates from each cow at the different samples stages (S1, S2 and S3) in these two herds only, and thereby influenced the interpretation of the longitudinal pattern of the results, especially with regard to persistence.

## Conclusion

We have identified 20 different CNS species that were associated with subclinical or clinical IMI in lactating cows on five US dairies across four states. There was variation in geographical distribution of the different species, and the three major species that occurred with the highest frequencies were *S. chromogenes*, *S. haemolyticus* and *S. simulans*. Repeated isolation of genetically indistinguishable *S. chromogenes* and *S. simulans* from the same udder quarter of multiple cows during consecutive sampling stages, or the occurrence of closely related or indistinguishable isolates from different cows on the same dairy could point at possible persistent infections; alternatively, further research is required to elucidate the potential for contagious transmission when similar isolates are found in different quarters or between cows with special attention to the fidelity of the tools used to assess isolates for similarity. Our findings are important in guiding the design of management strategies specific to the predominant CNS species cultured from cows on dairy farms, with considerations for prevalence, transmission pattern and possibility of persistent infections. Future research should investigate CNS species and risk factors for their occurrence, transmission, persistence with the goal of identifying appropriate control strategies. In addition, researching CNS species individually may further our knowledge about the role of these pathogens in IMI and their transmission patterns.

##  Supplemental Information

10.7717/peerj.6749/supp-1 Figure S1 Clustering of the CNS isolates based on the PFGE profiles The genetic relatedness and categorization of isolates into clades was interpreted based on the band profiles of *Staphylococcus hominis*. The different clades (A, B, C, D and E) were comprised of isolates with at least 50% similarity. Isolates with 100% similarity (B and C) were designated as genetically indistinguishable. The isolates were taken from herds in California (CA2), Iowa (IA), Minnesota (MN) and Wisconsin (WI). The phylogenetic tree was constructed based on the PFGE banding pattern using UPGMA algorithm. Udder quarters: LF, Left Front; RF, Right Front; RR, Right Rear; LR, Left Rear.

10.7717/peerj.6749/supp-2 Dataset S1 Dataset S1 The spreadsheet “RawData” contains the raw data and the spreadsheet “Dictionary” contains the definitions for acronyms used in the raw data.

## Additional Information and Declarations

### Data Availability

The following information was supplied regarding data availability: The raw data is available as Supplemental File.
