# Supplementary figures and images for: Molecular epidemiology of coagulase-negative Staphylococcus species isolated at different lactation stages from dairy cattle in the United States
Source: PeerJ. 2019 May 6;7:e6749. doi: 10.7717/peerj.6749 (PMC6507897; doi:10.7717/peerj.6749)

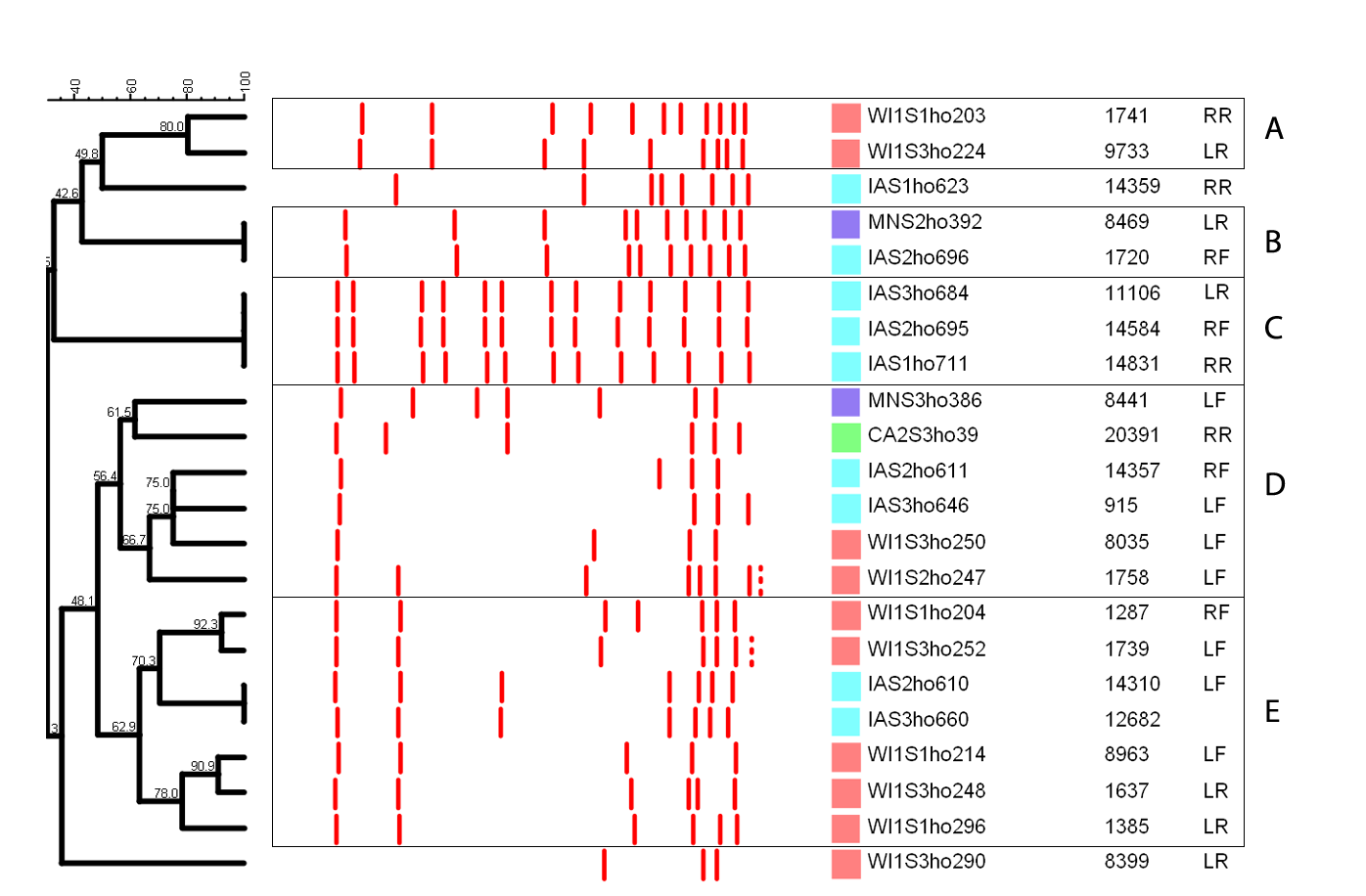

Supplement: Figure S1 — The genetic relatedness and categorization of isolates into clades was interpreted based on the band profiles of Staphylococcus hominis. The different clades (A, B, C, D and E) were comprised of isolates with at least 50% similarity. Isolates with 100% similarity (B and C) were designated as genetically indistinguishable. The isolates were taken from herds in California (CA2), Iowa (IA), Minnesota (MN) and Wisconsin (WI). The phylogenetic tree was constructed based on the PFGE banding pattern using UPGMA algorithm. Udder quarters: LF, Left Front; RF, Right Front; RR, Right Rear; LR, Left Rear. [file peerj-07-6749-s001.png]
